# Barettin Suppresses Pancreatic Ductal Adenocarcinoma Proliferation via Topoisomerase IIα Inhibition

**DOI:** 10.3390/md24060201

**Published:** 2026-06-07

**Authors:** Caleb A. Seekins, Monique R. Archuleta, Alexandria E. Evans, Julia Podgorski, Jerry E. Carr, Vishal Kaleeswaran, Kayla B. Nguyen, Matthew E. Flowers, Christopher Hulme, Todd W. Vanderah, Paco Cárdenas, John M. Streicher, Nam Y. Lee, Christopher Cartmell

**Affiliations:** 1 Department of Pharmacology, College of Medicine, University of Arizona, Tucson, AZ 85724, USA; 2 Department of Chemistry and Biochemistry, College of Science, University of Arizona, Tucson, AZ 85724, USA; 3 Department of Pharmacology & Toxicology, College of Pharmacy, University of Arizona, Tucson, AZ 85724, USA; 4 Comprehensive Center for Pain and Addiction, University of Arizona, Tucson, AZ 85724, USA; 5 Pharmacognosy, Department of Medicinal Chemistry, Uppsala University, 752 37 Uppsala, Sweden; 6 Museum of Evolution, Uppsala University, 752 37 Uppsala, Sweden; 7 Comprehensive Cancer Center, University of Arizona, Tucson, AZ 85724, USA; 8 Center for Applied Nano Bioscience and Medicine, College of Medicine, University of Arizona, Phoenix, AZ 85004, USA

**Keywords:** pancreatic ductal adenocarcinoma (PDAC), topoisomerase IIα, DNA gyrase, barettin, marine natural product

## Abstract

Pancreatic ductal adenocarcinoma (PDAC) remains a highly lethal malignancy with few therapeutic options. Topoisomerase IIα (TOPO2α) is frequently overexpressed in PDAC and is associated with poor clinical outcomes, yet current TOPO2α-directed therapies are constrained by limited efficacy and toxicity. Barettin, a brominated indole-containing diketopiperazine isolated from the marine sponge *Geodia barretti*, has not previously been evaluated against PDAC-relevant targets. Here, we identify barettin as a TOPO2α inhibitor using an integrated phenotypic, computational, and biochemical approach. Barettin exerts a cytostatic, non-toxic effect, selectively suppressing proliferation in a subset of PDAC models while showing reduced activity in others, revealing context-dependent efficacy and biological selectivity. Consistent with this, barettin inhibits TOPO2α-mediated DNA decatenation in vitro, demonstrating direct interference with enzyme activity. These findings support barettin as a selective inhibitor of a cancer-relevant proliferative pathway, uncovering a potential vulnerability in a subset of PDAC.

## 1. Introduction

Pancreatic ductal adenocarcinoma (PDAC) remains one of the most lethal human malignancies, with five-year survival rates persistently below ten percent and only marginal improvement over the past several decades [1,2]. According to GLOBOCAN statistics, 502k deaths per year are associated with pancreatic cancer; furthermore, with rising incidence and poor treatment options, PDAC is soon projected to be the second leading cause of cancer deaths [3,4]. This dismal prognosis reflects core features of the disease, including early metastatic dissemination [5], extensive intra- and intertumoral heterogeneity [6], late clinical presentation [7,8], and profound resistance to cytotoxic chemotherapy and radiation therapy [2,9,10]. Together, these factors underscore the urgent need for new therapeutic strategies that exploit functionally essential vulnerabilities in PDAC.

Topoisomerase II (TOPO2) is an established anticancer target that plays a central role in DNA replication [11], chromosome condensation and segregation, and maintenance of genomic stability in rapidly proliferating cells. Among its two human isoforms, TOPO2α is particularly relevant to cancer biology, as its expression is tightly linked to cell cycle progression and proliferation [12,13]. Notably, TOPO2α is frequently overexpressed in PDAC relative to normal pancreatic tissue and has been associated with high proliferative indices, increased genomic instability, and aggressive tumor behavior [14]. These observations have motivated efforts to therapeutically exploit TOPO2α in PDAC.

Several TOPO2-targeting agents have been evaluated in PDAC, either as monotherapies or in combination regimens. Classical TOPO2 poisons, such as natural products, etoposide [15,16] and doxorubicin [17,18,19] isolated from the North American Mayapple [20] (*Podophyllum peltatum*) and *Streptomyces peucetius* [21], respectively, have demonstrated clear on-target cytotoxicity with early-phase clinical trials in PDAC and related gastrointestinal malignancies. But despite modest clinical activity, these agents have failed to achieve durable responses or adoption as standard-of-care therapies in PDAC due to dose-limiting toxicities, limited therapeutic windows, and the emergence of resistance mechanisms [22]. These limitations highlight the need for alternative TOPO2-directed strategies, particularly those that may engage the enzyme through distinct or non-classical mechanisms. However, it is noteworthy that type II topoisomerases are found in all cellular domains of life, including bacteria, archaea, eukaryotes and even some viruses. In bacteria such as *Mycobacterium tuberculosis*, DNA Gyrase is a homolog of Topoisomerase IIa [23], leading to a potential drug repurposing avenue.

Marine natural products have emerged as a uniquely rich and underexplored source of anticancer drug leads [24,25,26,27]. The chemical and ecological pressures of the marine environment drive the evolution of structurally complex, highly bioactive secondary metabolites that are rarely found in terrestrial organisms [24,28]. Several clinically important anticancer agents illustrate this potential: didemins, isolated from Mediterranean tunicates [29,30]; cytarabine [31], originally inspired by nucleosides isolated from the Caribbean sponge *Tectitethya crypta*; trabectedin [32,33], derived from the sea squirt *Ecteinascidia turbinata*; eribulin, a synthetic analog of halichondrin B from the Japanese marine sponge *Halichondria okadai*; and eleutherobin [26], a microtubule-stabilizing diterpene isolated from the soft coral *Eleutherobia* sp. [34]. These compounds target fundamental cellular processes such as DNA replication and microtubule dynamics, validating marine ecosystems as a proven pipeline for oncology therapeutics.

**Figure 1:**
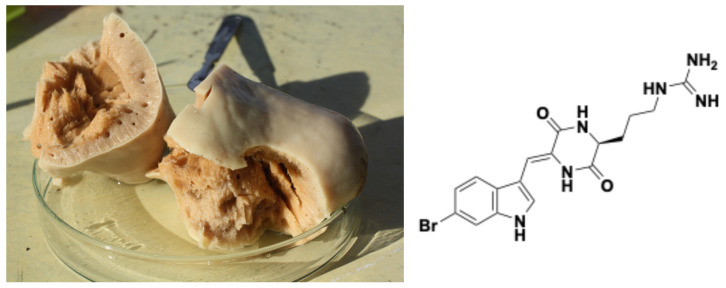
Image of the marine sponge *Geodia barretti* (**left**) with the structure of barettin (**right**).

Barettin (Figure 1) was originally isolated in 1986 from the marine deep-sea sponge *Geodia barretti* and subsequent investigations revealed its pronounced antibiofouling activity [35], with an ED_50_ of 0.9 μM [36]. More recently, we have demonstrated barettin to be an inverse agonist of 5HT2a, resulting in antinociceptive properties in an in vivo chemotherapy-induced peripheral neuropathy model [37]. Structurally, barettin is distinguished by a β-unsaturated brominated tryptophan residue and an arginine moiety cyclized into a 2,5-diketopiperazine (DKP) core. This chemically compact yet highly functionalized scaffold is of particular interest from a drug discovery perspective, as DKPs are known to exhibit favorable physicochemical properties and serve as privileged structures amenable to medicinal chemistry optimization [38,39].

Herein, using cell-based assays, molecular docking, and in vitro decatenation approaches, we identify barettin as a previously unrecognized inhibitor of TOPO2α activity. These findings position barettin as a promising natural product scaffold for further investigation as a non-classical TOPO2α modulator and suggest potential utility in targeting proliferative vulnerabilities in cancer and infectious diseases.

## 2. Results and Discussion

### 2.1. Anticancer Activity of Barettin

**Figure 2:**
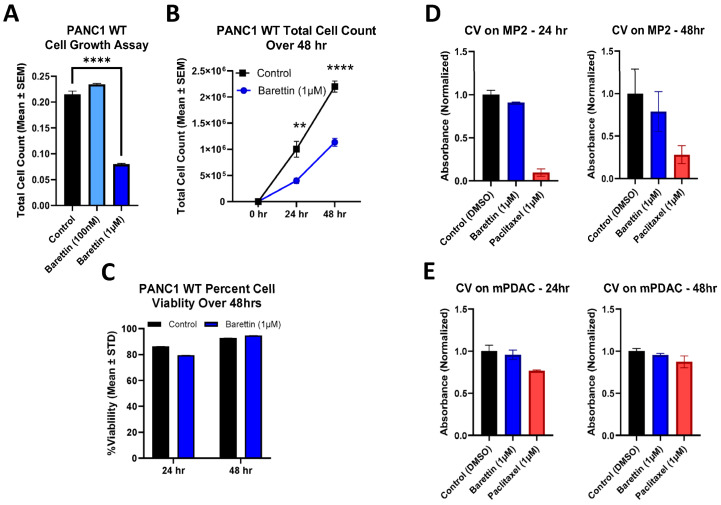
Baretin inhibits PANC1 cell growth but not cell viability and has no significant effect on MP2 or MPDAC cells. (**A**) Treatment of 1 µM Barretin produces a significant reduction in cell proliferation compared to control, while 100 nM has no effect. (**B**) Barretin-induced reduction in cell proliferation is compounded over time, with a significant decline at both 24 h and 48 h time points. (**C**) Barretin does not induce cell death in PANC1 wild-type cells with 24 h or 48 h exposure. (**D**) Barretin has no effect on MP2 cell viability based on the crystal violet assay with neither 24 h nor 48 h exposure, and is less effective than paclitaxel, a known chemotherapeutic. (**E**) Similarly, Barretin has no effect on mPDAC cell viability based on the crystal violet assay with either 24 h or 48 h exposure. ** *p* = 0.01, **** *p* < 0.0001 vs. same time point Control group using one-way ANOVA with Tukey’s multiple comparisons. Data is reported as the mean ± SEM. The growth assay was performed in quadruplicate, with Crystal Violet performed in triplicate.

To evaluate the anticancer activity of barettin, we first examined its effects on the growth of a prominent human PDAC cell line, Panc-1. Using direct cell counting, treatment with barettin at 1 μM resulted in more than 50% reduction in total cell number over 48 h, whereas 100 nM had no measurable effect (Figure 2A). A comparable reduction in growth was observed at both 24 and 48 h following 1 μM treatment (Figure 2B). Notably, this suppression occurred without a decrease in viability between treated and control groups at both timepoints (Figure 2C), suggesting that barettin inhibits proliferation growth through a cytostatic, rather than cytotoxic mechanism.

To define the selectivity of this response, we extended these studies to another established human PDAC cell line, MiaPaCa-2 (MP2), and mouse PDAC cells derived from a genetically engineered model (mPDAC), representing distinct proliferative and therapy-response states. In contrast to Panc-1, barettin (1 μM) produced only modest growth inhibition in MP2 cells (Figure 2D) despite their robust sensitivity to paclitaxel, a mitotic inhibitor, while mPDAC cells (Figure 2E) were largely unresponsive to both agents, consistent with a therapy-refractory phenotype as reported previously.

These findings support barettin as a cytostatic, non-toxic inhibitor of PDAC cell proliferation with clear context-dependent activity. The absence of cell death in Panc-1 cells, together with prior evidence of minimal cytotoxicity of barettin at substantially higher doses in multiple other cell types [37,40], indicates that the observed growth suppression reflects a specific biological effect rather than nonspecific toxicity. Importantly, these models capture a range of PDAC behaviors, with Panc-1 cells strongly dependent on proliferation, MP2 cells remaining sensitive to some mitotic inhibitors, and mPDAC cells exhibiting a more resistant, stress-adapted phenotype. Accordingly, here the biological selectivity of barettin highlights its potential as a therapeutic for a subset of PDAC.

### 2.2. Identifying Topoisomerase II Activity

**Figure 3:**
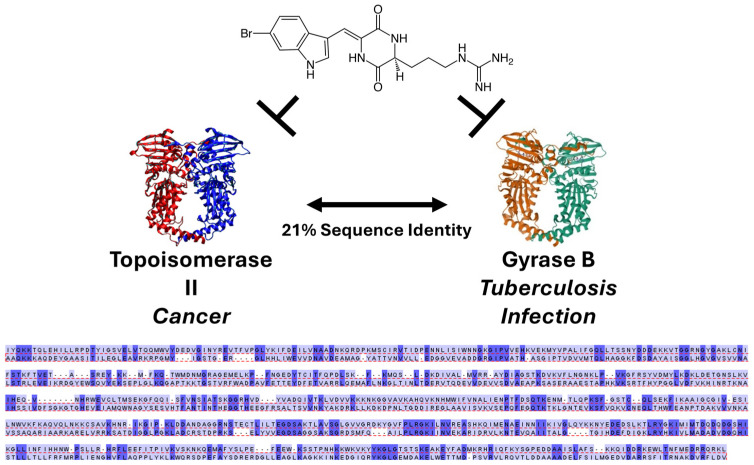
Cobalt comparison of human topoisomerase II against *Mycobacterium tuberculosis* DNA Gyrase, displaying 21% sequence identity.

To identify the molecular target of barettin, we considered prior work implicating indole-containing small molecules in the modulation of type II topoisomerases. Notably, Akabayov et al. demonstrated that indole-based compounds can selectively inhibit DNA gyrase in *Mycobacterium tuberculosis*, suggesting a potential link between the indole scaffold of barettin and topoisomerase targeting [41]. Supporting this, Lewis et al. reported that speirobactin, a structurally related natural product, potently inhibits DNA gyrase A, further supporting indole-containing natural products as modulators of type II topoisomerases [42]. DNA gyrase is a bacterial type II topoisomerase that shares approximately 21% sequence similarity with human topoisomerase IIα (TOPO2α) (Figure 3), with conservation of key catalytic and ATP-binding domains. Together, these observations provide a plausible structural and functional basis for testing whether barettin inhibits TOPO2α activity.

### 2.3. Molecular Target Identification: Docking

Molecules that inhibit TOPO2α can be broadly categorized into two classes: TOPO2α poisons (e.g., etoposide [16,43], doxorubicin [22], etc.) and TOPO2α catalytic inhibitors [44] (e.g., dexrazoxane [18], topobexin [45], etc.). TOPO2α poisons work by forming ternary complexes with TOPO2α and DNA, trapping the enzyme to the 5′ end of DNA double-strand breaks and preventing DNA religation [46]. In contrast, the catalytic inhibitors decrease the ATPase domain activity and constrain TOPO2α to a closed-clamp formation [47]. Unsurprisingly, the binding sites for these inhibitors differ depending on their mode of action.

**Figure 4:**
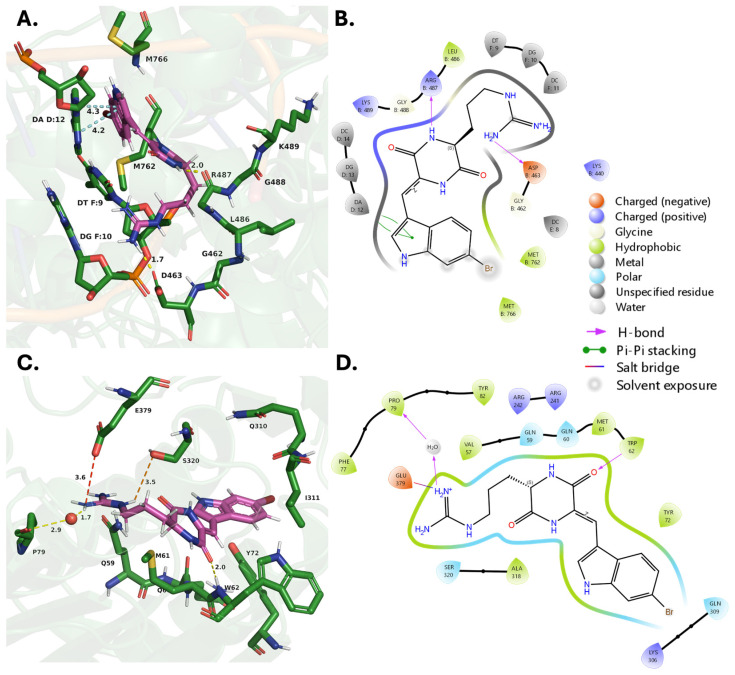
Results from molecular docking experiments. Key interactions are depicted as follows: salt bridges (red), hydrogen bonds (yellow, strong; orange, weak), and π–π interactions (cyan). (**A**) Docking pose of barettin (magenta) in the etoposide binding site of human TOPO2α (PDB: 5GWK-barettin glidescore = −7.79 kcal/mol (third-ranked pose). (**B**) 2D ligand interaction diagram corresponding to the pose shown in (**A**). (**C**) Docking pose of barettin in the topobexin binding site within the ATPase domain of TOPO2α (PDB: 9BQB-barettin glidescore = −6.70 kcal/mol (top-ranked pose)). (**D**) 2D map of the interactions formed by barettin in the pose depicted in (**C**).

To gain insight into the potential mode of TOPO2α inhibition by barettin, molecular docking experiments were performed using the Glide-SP protocol [48]. The published crystal structure of TOPO2α bound to etoposide (PDB: 5GWK) was used to assess barettin’s potential as a TOPO2α poison. Docking results indicate that barettin forms hydrogen bonds with residues D463 and R487, along with π-stacking interactions with the DNA base pairs, as shown in Figure 4. These interactions closely resemble those formed by etoposide in its binding site in 5GWK (Supplementary Figure S4).

Unfortunately, no experimental co-crystal structures of human TOPO2α and dexrazoxane are currently available in the Protein Data Bank. Hence, to model barettin’s potential activity as a catalytic inhibitor, the high-resolution structure of the human TOPO2α ATPase domain bound to topobexin (PDB: 9BQB) was used [46]. In this model, barettin forms a hydrogen bond with W62, analogous to topobexin, and additionally interacts with P79 through an active-site water molecule. Apart from this, the ligand also participates in a salt-bridge interaction with E379 and a weak hydrogen-bond interaction with S320, as depicted in Figure 4.

The scores generated through the Glide SP protocol for the poses depicted in Figure 4 are given below. The 10 poses generated were manually inspected and only those that formed key interactions were considered. Key interactions were determined from the interactions formed by reference ligands, including etoposide in 5GWK and topobexin in 9BQB.

Although the docking results suggest that barettin may act as a TOPO2α poison based on its similarity to the etoposide binding mode, it should be noted that topobexin occupies a binding site distinct from that of dexrazoxane [46]. Further, barettin and dexrazoxane share a high degree of structural similarity as both have the diketopiperazine moiety. Taken together, these observations suggest that the mechanism of action of barettin on TOPO2α remains ambiguous.

### 2.4. Decatenation Assay

**Figure 5:**
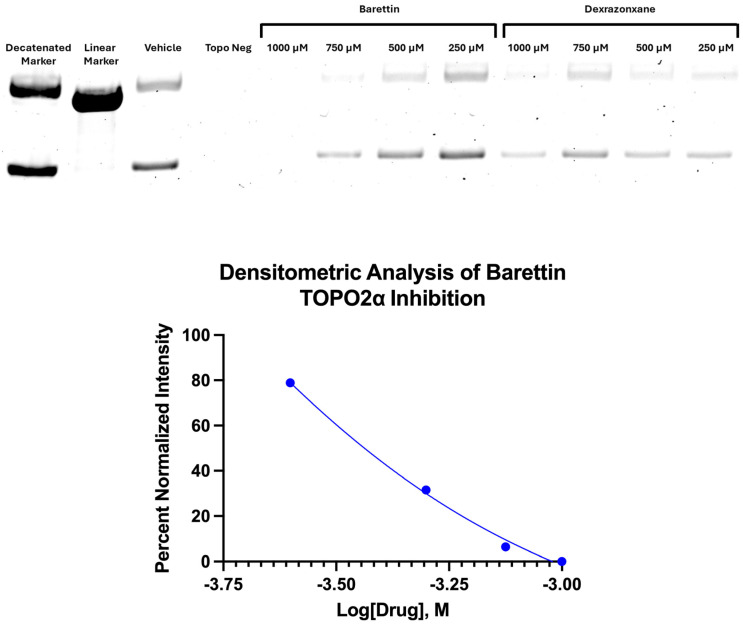
(**Top**) Barettin acts via blocking TOPO2α in vitro in the decatenation assay. Barettin produces a dose-dependent reduction in decatenated kDNA bands via a blockade of TOPO2α. This effect is similar to the effects produced by Dexrazoxane, a strong catalytic inhibitor of topoisomerase. As shown here as a loss of the decatenated marker in an agarose gel. (**Bottom**) Densitometric analysis of barettin for inhibition of TOPO2α with a calculated IC50 of 108.3 uM and an R^2^ of 0.9960.

To directly assess whether barettin affects the catalytic activity of type II topoisomerase, we employed a kDNA decatenation assay (Figure 5) using the Topoisomerase II Drug Screening Kit (TopoGEN). This widely used biochemical assay monitors the ability of human Topo IIα to decatenate interlocked kinetoplast DNA (kDNA) networks in vitro and resolve them into discrete DNA minicircles that migrate into an agarose gel matrix. In the absence of enzyme activity, the large catenated kDNA networks remain trapped in the well and do not enter the gel; conversely, active Topo IIα generates decatenated minicircular DNA species that are readily visualized by ethidium bromide staining upon electrophoresis.

In our experiments, reactions containing purified human Topo IIα in the presence of vehicle control produced robust decatenation of kDNA, as evidenced by the appearance of discrete DNA bands corresponding to open circular and relaxed minicircle products that migrated into the gel. The known catalytic inhibitor dexrazoxane was included as a positive control to validate assay performance; under these conditions, dexrazoxane effectively suppressed the formation of decatenated products, resulting in the retention of kDNA in the well and the loss of decatenated bands.

When barettin was incubated with Topo IIα under identical assay conditions, we observed a similar pattern to dexrazoxane, with a marked loss of decatenated kDNA bands and a corresponding accumulation of catenated kDNA retained near the origin of electrophoretic migration. This shift in the DNA banding pattern indicates that barettin inhibits the decatenase activity of Topo IIα, preventing the release of minicircle products and thereby blocking the conversion of high-molecular-weight kDNA networks into lower-molecular-weight species.

Taken together, these data demonstrate that barettin interferes with the catalytic function of human TOPO2α in vitro. The observed loss of decatenated DNA products upon treatment with barettin, comparable to the known inhibitor dexrazoxane, supports the conclusion that barettin is capable of inhibiting Topo IIα-mediated DNA decatenation. This biochemical inhibition aligns with the phenotypic growth suppression observed in Panc-1 cells and provides a mechanistic basis for further exploration of barettin as a scaffold for targeting type II topoisomerase activities. Future studies will be required to dissect whether barettin acts as a catalytic inhibitor, a poison, or a novel modulator of enzyme dynamics.

## 3. Materials and Methods

### 3.1. Isolation of Barettin

Three specimens of *G. barretti* were collected by Karin Steffen on board the research vessel Hans Brattstrøm, with a triangular dredge, on 8–9 September 2016, at 250–330 m depth, in the Korsfjord and Langenuen, south of Bergen, Norway. Specimens were identified on board by sponge taxonomist Prof. H. T. Rapp and frozen upon collection. Specimens were sent frozen to Uppsala University, Sweden, where they were freeze-dried before being sent to the University of Arizona. The specimens used in this paper are identified with the following museum accession numbers: Uppsala Zoological Museum Collection UPSZMC 195453, UPSZMC 195454, and UPSZMC 195455. Freeze-dried sponge extract was placed on filter paper in a funnel and rinsed with dichloromethane (DCM) to wash away lipid contaminants in the sample [37,38,39]. After the sample was rinsed with DCM, the freeze-dried extract was washed with a solution of 60% acetonitrile. These washes were analyzed using LC-MS and were later combined. The sample was concentrated to a final volume of 1.5 mL. Barettin was then purified using RP-HPLC on a Phenomenex Luna C18 (5-micron, 250 × 21.2 mm) column with UV detection at 234 nm. The compound was separated using a linear gradient, beginning with 95% solvent A (0.1% formic acid in Milli-Q water) and 5% solvent B (acetonitrile) and over 40 min advancing to 40% solvent B. Then, solvent B was increased to 95% over 15 min, held for 5 min, and returned to the starting conditions. The retention time for barettin was determined to be 37 min, and LC-MS confirmed its presence; further analysis was conducted using NMR spectroscopy.

***(Z)-1-(3-(5-((6-bromo-1H-indol-3-yl) methylene)-3,6-dioxopiperazin-2-yl) propyl)guanidine* ^1^H NMR (500 MHz, DMSO)** δ 11.82 (s, 1H), 9.65 (s, 1H), 8.40 (d, *J* = 2.7 Hz, 1H), 7.96 (d, *J* = 2.7 Hz, 1H), 7.65–7.56 (m, 3H), 7.23 (d, *J* = 8.5 Hz, 1H), 6.96 (s, 1H), 4.05 (td, *J* = 5.7, 2.6 Hz, 1H), 3.12 (q, *J* = 6.7 Hz, 2H), 1.74 (dd, *J* = 9.5, 5.6 Hz, 2H), 1.55 (h, *J* = 6.5 Hz, 2H). **^13^C NMR (126 MHz, DMSO)** δ 167.2 (CO), 161.2 (CO), 157.2 (C(NH)_2_NH_2_,) 137.0 (C), 127.8 (CH), 126.5 (C), 123.5 (CH), 123.2 (CH), 120.5 (CH), 115.2 (C), 114.9 (CBr), 108.6 (CH), 107.5 (C), 55.1 (CH), 40.5 (CH_2_), 31.7 (CH2), 24.5 (CH_2_). **MS (ESI)** *m*/*z* 421 (100) [M(^81^Br) + H]^+^, 419 (100) [M(79Br) +H]^+^; **HRMS (FTMS + p ESI):** *m*/*z* calculated for: C_17_H_20_BrN_6_O [M(^79^Br) + H]^+^: 419.0826; found: 419.0809.

### 3.2. Growth Assay

The antiproliferative effects of barettin were evaluated using human pancreatic cancer cell lines Panc-1 and MiaPaCa-2, as well as a murine pancreatic ductal adenocarcinoma cell line (mPDAC). Cells were maintained under standard culture conditions at 37 °C in a humidified incubator with 5% CO_2_ and cultured in their recommended growth media supplemented with 10% fetal bovine serum and 1% penicillin–streptomycin.

For growth assays, cells were seeded into 12-well plates and allowed to adhere overnight. Following attachment, cells were treated with barettin dissolved in DMSO at the indicated concentrations. Vehicle controls received an equivalent volume of DMSO, which was kept constant across all conditions. Each treatment condition was performed in quadruplicate wells.

Cells were incubated with the compound for 48 h, after which cell growth was quantified by cell counting. Briefly, cells were washed with PBS, detached using trypsin–EDTA, and resuspended in fresh medium. Viable cells were counted using a hemocytometer and trypan blue exclusion. Growth was calculated relative to vehicle-treated controls.

### 3.3. Crystal Violet Assay

PDAC cells were plated in 12-well plates in quadruplicate. At the indicated time points, cells were fixed with 4% paraformaldehyde for 15 min, washed once with water, and stained with 0.1% crystal violet for 30 min. Cells were then washed three times with PBS, air-dried for 30 min, and destained with a solution containing 10% acetic acid, 50% methanol, and 40% water for 15 min. Absorbance was measured at 590 nm using a microplate reader.

### 3.4. Docking

Molecular docking was performed using Schrödinger Maestro Release 2026-1. The protein structures were procured from the Protein Data Bank (PDB: 5GWK, 9BQB) and were prepared using the Protein Preparation Workflow with default parameters. Three-dimensional coordinates for the ligand barettin were obtained using LigPrep, and the Receptor Grid Generation tool was used to generate a 20 × 20 × 20 Å grid around the binding site of the inhibitors. Barettin was then docked using the Glide-SP protocol with default parameters, asking for up to 10 poses. The binding poses were analyzed and exported as a .pdb file. PyMOL 3.1.6.1 was used for visualizing the data and generating images. 

### 3.5. Decatenation Assay

Topoisomerase II–mediated DNA decatenation was evaluated using a Topoisomerase II Drug Screening Kit (TopoGEN, Inc., Columbus, OH, USA) according to the manufacturer’s instructions. This assay measures the ability of purified human topoisomerase IIα to decatenate kinetoplast DNA (kDNA) networks into free minicircular DNA that can be resolved by agarose gel electrophoresis.

Reaction mixtures (20 μL total volume) contained kDNA substrate, assay buffer supplied with the kit, ATP, and purified human Topoisomerase IIα enzyme. Barettin was dissolved in DMSO and added to reactions at the indicated concentrations. Dexrazoxane was used as a positive control inhibitor of Topoisomerase IIα, while vehicle (DMSO) served as a negative control. The final concentration of DMSO was kept constant across all reactions.

Reactions were incubated at 37 °C for 30 min and terminated by the addition of stop buffer containing SDS and proteinase K to digest enzyme–DNA complexes. Samples were then resolved by electrophoresis on a 1% agarose gel prepared in TAE buffer and stained with ethidium bromide. DNA bands were visualized under UV illumination and imaged using a gel documentation system.

Successful decatenation of kDNA by Topoisomerase IIα was indicated by the appearance of decatenated minicircle DNA migrating into the gel, whereas inhibition of enzyme activity resulted in retention of catenated kDNA networks near the loading wells. All experiments were performed in at least three independent replicates. Gel imaged with an Azure Biosystems Sapphire biomolecular imager. The resulting image bands were quantified using ImageJ version 1.54t (National Institutes of Health). The kDNA intensities were normalized to a vehicle control present on the same gel.

## 4. Conclusions

Here, we identify barettin as a bioactive marine natural product with previously unrecognized effects on pancreatic cancer cells. Barettin exerts strong growth inhibition in Panc-1 cells without evident toxicity, consistent with a cytostatic mechanism. This activity in an aggressive PDAC model highlights the intrinsic biological potency of barettin, while its reduced activity in MP2 cells and mouse PDAC cells likely highlights a context-dependent, biologically selective effect within a subset of PDAC.

We also evaluated barettin in a biochemical decatenation assay using purified human topoisomerase IIα. Barettin inhibited enzyme-mediated decatenation of kinetoplast DNA, resulting in a marked loss of decatenated DNA products on agarose gels, comparable to the effect observed with the known topoisomerase II inhibitor dexrazoxane. These results demonstrate that barettin can directly interfere with the catalytic activity of a key DNA-processing enzyme in vitro.

Collectively, these findings expand the biological profile of barettin beyond its previously reported antifouling and antioxidant activities and establish it as a marine natural product capable of modulating cancer-relevant cellular processes. Importantly, our recent work demonstrated that barettin also possesses nonopioid antihyperalgesic activity mediated through 5HT2A inverse agonism without hallucinogenic effects, highlighting its potential as a multifunctional scaffold with relevance to both cancer biology and supportive care applications, combining the treatment of chemotherapy-induced peripheral neuropathy whilst also preventing PDAC proliferation [37]. Given its compact, chemically tractable scaffold and low reported nonspecific cytotoxicity in prior studies, barettin represents a promising starting point for further mechanistic, structural, and medicinal chemistry investigations. This work underscores the continued value of marine natural products as sources of structurally unique molecules with potential relevance to cancer drug discovery.

## Data Availability

All data required to interpret these findings are contained within the manuscript or Supporting data. NMR data ^1^H and ^13^C can be viewed using the NP-MRD, (58) NP-CARD ID: NP0068647 with MS data available upon request to the corresponding author.

## Supplementary Materials

The following supporting information can be downloaded at https://www.mdpi.com/article/10.3390/md24060201/s1, NMR and LCMS data, as well as the molecular docking of etoposide.
