# Supplementary material for: Barettin Suppresses Pancreatic Ductal Adenocarcinoma Proliferation via Topoisomerase IIα Inhibition
Source: Mar Drugs. 2026 Jun 7;24(6):201. doi: 10.3390/md24060201 (PMC13302744; doi:10.3390/md24060201)
Supplement: Supplementary file 1 [file marinedrugs-24-00201-s001.zip › marinedrugs-4342039-supplementary.pdf]

Supporting Information.

Barettin Suppresses Pancreatic Ductal Adenocarcinoma Proliferation via Topoisomerase IIα Inhibition

Caleb A. Seekins [1,†], Monique R. Archuleta [1,†], Alexandrea E. Evans [2], Julia Podgorski [1], Jerry E. Carr [1], Vishal Kaleeswaran [2], Kayla B. Nguyen [1], Matthew E. Flowers [1], Christopher Hulme [2,3], Todd W. Vanderah [1,4], Paco Cárdenas [5,6], John M. Streicher [1,4], Nam Y. Lee [1,7] and Christopher Cartmell [1,4,7,8,*]

[1] Department of Pharmacology, College of Medicine, University of Arizona, Tucson, AZ, USA; 85724

[2] Department of Chemistry and Biochemistry, College of Science, University of Arizona, Tucson, AZ, USA; 85724

[3] Department of Pharmacology & Toxicology, College of Pharmacy, University of Arizona, Tucson, AZ, USA; 85724

[4] Comprehensive Center for Pain and Addiction, University of Arizona, Tucson, AZ, USA, 85724

[5] Pharmacognosy, Department of Medicinal Chemistry, Uppsala University, Sweden; 752 37

[6] Museum of Evolution, Uppsala University, Sweden752 37

[7] University of Arizona Comprehensive Cancer Center, University of Arizona, Tucson, AZ, USA, 85724

[8] Center for Applied Nano Bioscience and Medicine, College of Medicine, University of Arizona, Phoenix, AZ, USA, 85004

* Correspondence: cartmell@arizona.edu

† These authors contributed equally to this work.

^{1}H NMR of Barettin (Figure S1) 2

^{13}C NMR of Barettin (Figure S2) 3

LC and HRMS traces (Figure S3) 4

2D Ligand interaction diagram of etoposide in human TOP2A (PDB:5GWK) 5

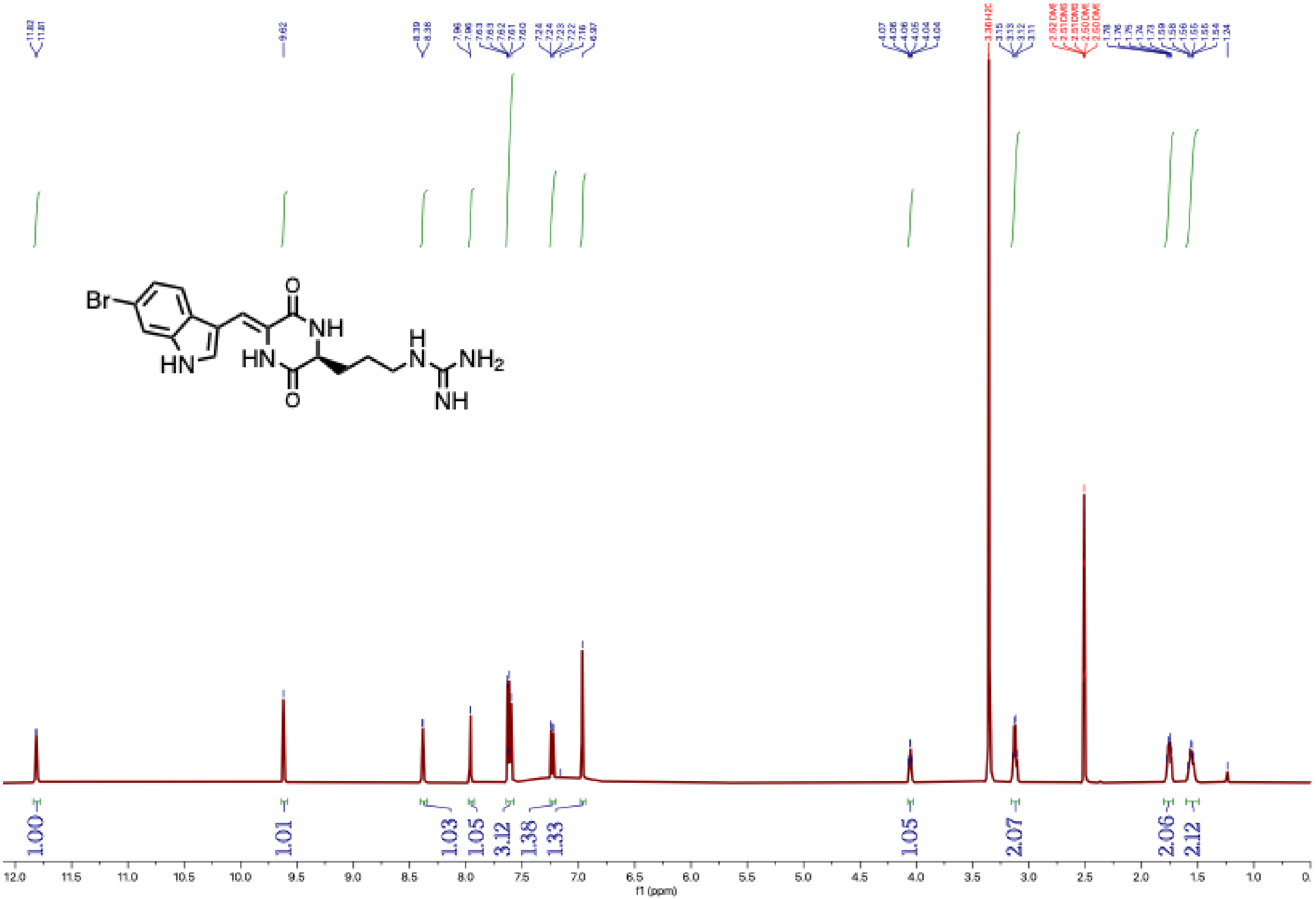

Figure S1: ^{1}H NMR of Barettin

^{1}H NMR (500 MHz, DMSO) δ 11.82 (s, 1H), 9.65 (s, 1H), 8.40 (d, *J* = 2.7 Hz, 1H), 7.96 (d, *J* = 2.7 Hz, 1H), 7.65 – 7.56 (m, 3H), 7.23 (d, *J* = 8.5 Hz, 1H), 6.96 (s, 1H), 4.05 (td, *J* = 5.7, 2.6 Hz, 1H), 3.12 (q, *J* = 6.7 Hz, 2H), 1.74 (dd, *J* = 9.5, 5.6 Hz, 2H), 1.55 (h, *J* = 6.5 Hz, 2H).

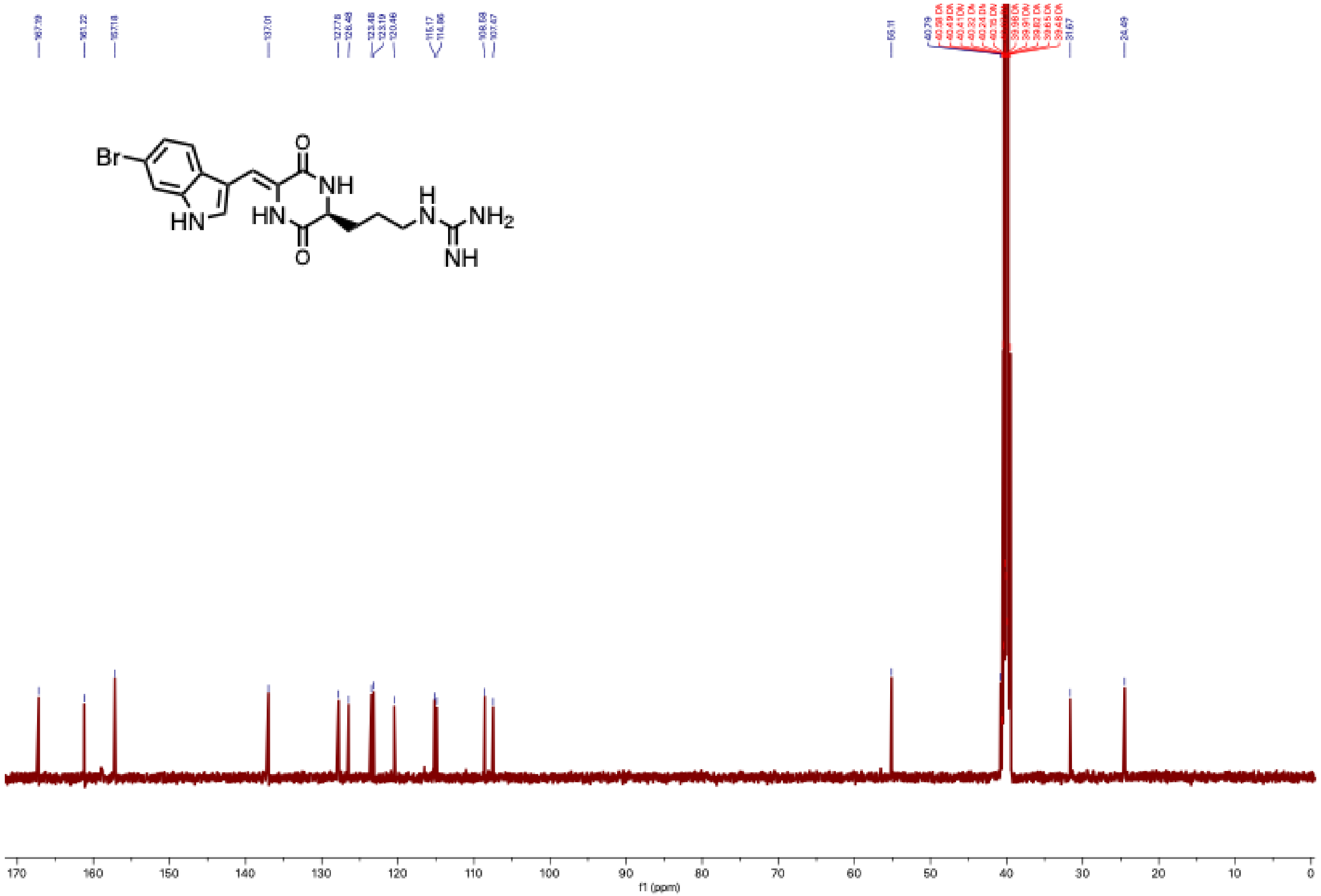

Figure S2: ^{13}C NMR of Barettin.

^{13}C NMR (126 MHz, DMSO) δ 167.2 (CO), 161.2 (CO), 157.2 ($C(NH)_2NH_2$,) 137.0 (C), 127.8 (CH), 126.5 (C), 123.5 (CH), 123.2 (CH), 120.5 (CH), 115.2 (C), 114.9 (CBr), 108.6 (CH), 107.5 (C), 55.1 (CH), 40.5 (CH_2), 31.7 (CH2), 24.5 (CH_2).

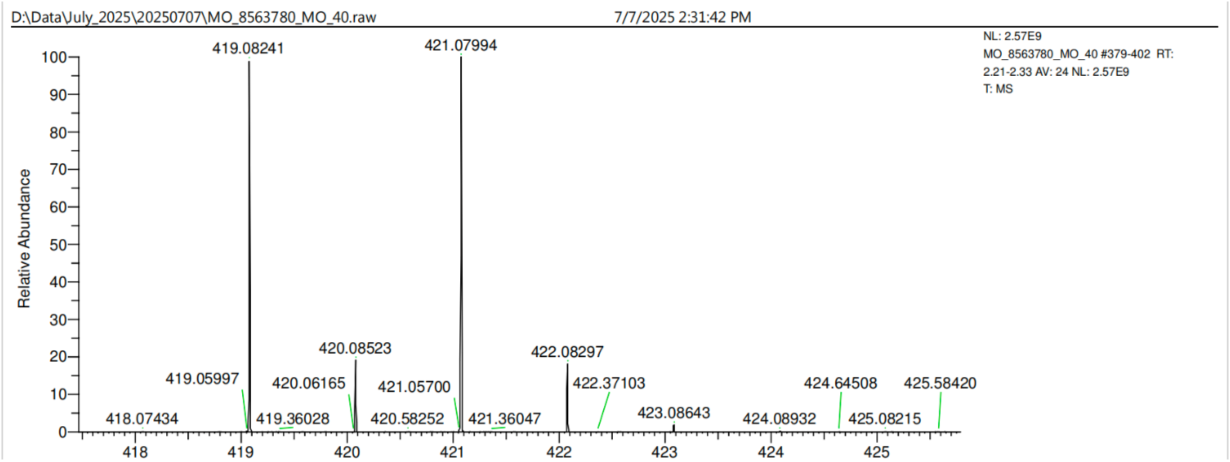

***Figure S3*:** LC and HRMS traces

MS (ESI) *m/z*, 419 (100) [M (^{79}Br) +H]$^+$, 421 (100) [M(^{81}Br) + H] $^+$; **HRMS (FTMS + p ESI):** m/z calculated for: $C_{17}H_{20}BrN_6O$ [M (^{79}Br) + H]$^+$: 419.0826; found: 419.0824.

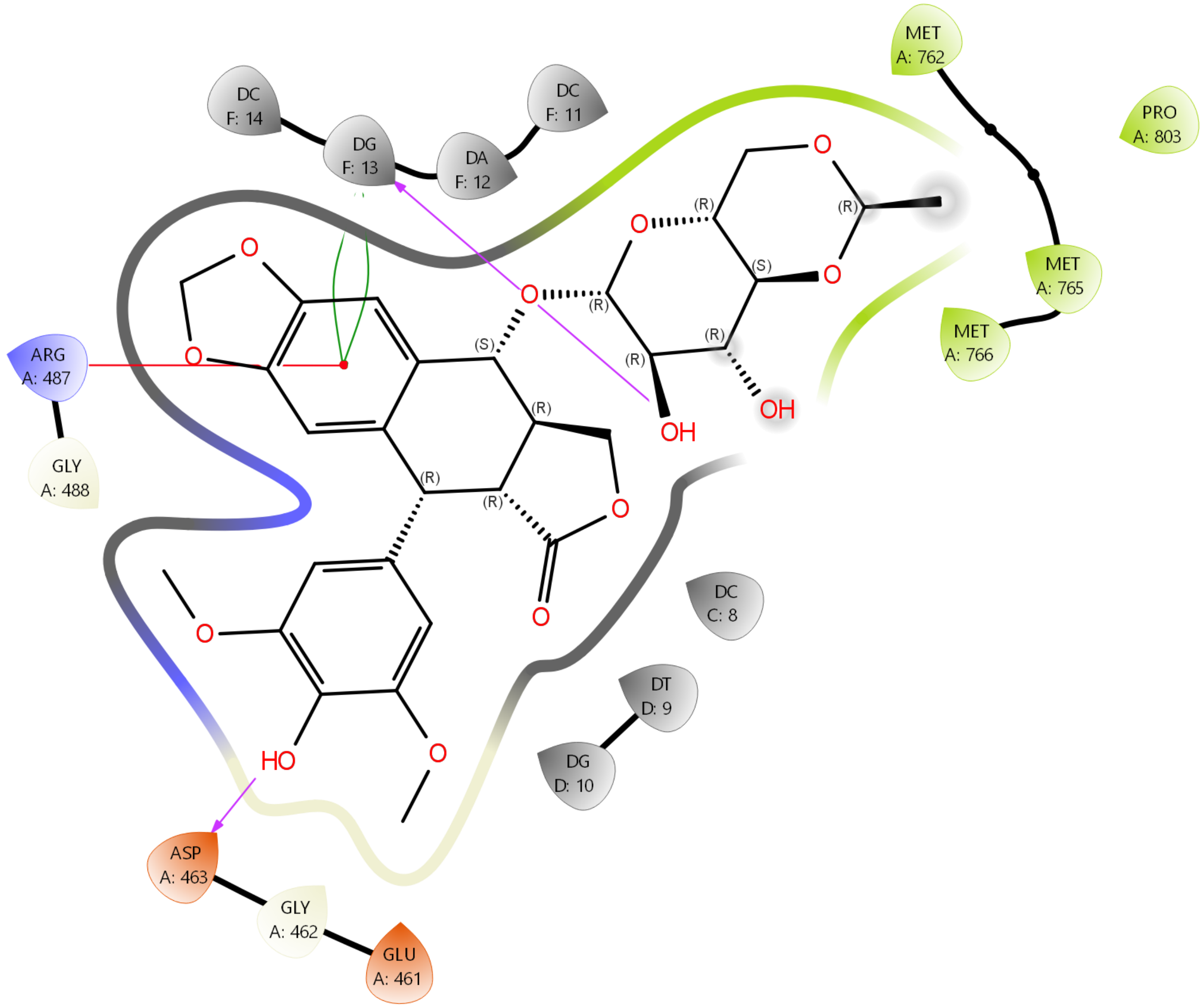

*Figure S4***:** 2D Ligand interaction diagram of etoposide in human TOP2A (PDB:5GWK)
